# Efficacy of transcranial direct-current stimulation (tDCS) in women with provoked vestibulodynia: study protocol for a randomized controlled trial

**DOI:** 10.1186/s13063-016-1366-5

**Published:** 2016-05-14

**Authors:** Annie Morin, Guillaume Léonard, Véronique Gougeon, Guy Waddell, Yves-André Bureau, Isabelle Girard, Mélanie Morin

**Affiliations:** School of Rehabilitation, Faculty of Medicine and Health Sciences, Université de Sherbrooke, 3001 12th Avenue North, Sherbrooke, Québec Canada; Department of Obstetrics Gynecology, Faculty of Medicine and Health Sciences, Université de Sherbrooke, 3001 12th Avenue North, Sherbrooke, Québec Canada

**Keywords:** Randomized clinical trial, Vestibulodynia, Transcranial direct-current stimulation, Chronic pain, Sexual dysfunction, Psychological distress, Treatment outcome, Dyspareunia

## Abstract

**Background:**

Provoked vestibulodynia is the most common form of vulvodynia. Despite its high prevalence and deleterious sexual, conjugal, and psychological repercussions, effective evidence-based interventions for provoked vestibulodynia remain limited. For a high proportion of women, significant pain persists despite the currently available treatments. Growing evidence suggests that the central nervous system (CNS) could play a key role in provoked vestibulodynia; thus, treatment targeting the CNS, rather than localized dysfunctions, may be beneficial for women suffering from provoked vestibulodynia. In this study, we aim to build on the promising results of a previous case report and evaluate whether transcranial direct-current stimulation, a non-invasive brain stimulation technique targeting the CNS, could be an effective treatment option for women with provoked vestibulodynia.

**Methods/design:**

This single-center, triple-blind, parallel group, randomized, controlled trial aims to compare the efficacy of transcranial direct-current stimulation with sham transcranial direct-current stimulation in women with provoked vestibulodynia. Forty women diagnosed with provoked vestibulodynia by a gynecologist, following a standardized treatment protocol, are randomized to either active transcranial direct-current stimulation treatment for ten sessions of 20 minutes at an intensity of 2 mA or sham transcranial direct-current stimulation over a 2-week period. Outcome measures are collected at baseline, 2 weeks after treatment and at 3-month follow-up. The primary outcome is pain during intercourse, assessed with a numerical rating scale. Secondary measurements focus on the sexual function, vestibular pain sensitivity, psychological distress, treatment satisfaction, and the patient’s global impression of change.

**Discussion:**

To our knowledge, this study is the first randomized controlled trial to examine the efficacy of transcranial direct-current stimulation in women with provoked vestibulodynia. Findings from this trial are expected to provide significant information about a promising intervention targeting the centralization of pain in women with provoked vestibulodynia.

**Trial registration:**

Clinicaltrials.gov, NCT02543593. Registered on September 4, 2015.

**Electronic supplementary material:**

The online version of this article (doi:10.1186/s13063-016-1366-5) contains supplementary material, which is available to authorized users.

## Background

Chronic vulvar pain represents a major health concern for women. Vulvodynia is a highly neglected chronic pain condition, affecting nearly 10 % of the female population [1]. Suspected to be the foremost cause of pre-menopausal vulvodynia, provoked vestibulodynia (PVD) is characterized by an acute recurrent pain located at the vulvar vestibule (i.e., the vaginal entrance) in response to pressure application or attempted vaginal penetration [2]. It has been revealed that PVD disrupts personal lives, severely affects sexual function, and negatively impacts the quality of life [3, 4]. PVD has also been related to relationship problems and psychological distress [5]. Poorly understood and often misdiagnosed or ignored, PVD pain leads to a high personal cost for patients and substantial financial cost for society. Women with PVD often multiply their medical visits hoping to find relief and rely mainly on non-evidence-based, ineffective interventions [6].

Many empirical treatment options are proposed to women with PVD. Firstly, localized interventions such as topical lidocaine application [7], pelvic floor physical therapy [8], biofeedback technique [9], Botox® injection [10], topical use of estradiol and testosterone compounds [11], and vestibulectomy (surgery) [12] can be offered. As a second option, psychotherapeutic interventions can be proposed, which include cognitive behavioral therapy (CBT) focusing on reducing pain and improving sexual function [13]; hypnotherapy [14]; or acupuncture [15]. In addition, the use of systemic treatments, such as tricyclic antidepressants [16], anticonvulsant medication and selective serotonin reuptake inhibitors (SSRIs) [17], can also be prescribed. Despite this wide variety of treatment options, a high proportion of women with PVD are refractory to conventional treatments [12, 17, 18], highlighting the need for novel approaches.

Recently, alterations in central pain mechanisms have been suggested to play a key role in PVD, potentially explaining the limited success obtained in some patients, with treatments targeting the pain locally in the area of the vestibule. Central sensitization has been observed in many chronic pain conditions, including chronic pelvic pain [19]. While the heightened sensitivity of peripheral pain receptors following local trauma or infection usually resolves with time, in chronic pain, this hypersensitivity is sustained and amplified by an extensive central neural network that includes the spinal dorsal horn, limbic system, and cortical structures [20]. Recent studies suggest that the pathophysiology of PVD is not only limited to the vulvar vestibule but also involves central pain processing alterations similarly to other chronic pain conditions [21]. In support of such central alterations, women with PVD have been shown to be more sensitive in areas other than the genital regions compared to controls (lower pain threshold) [21–23]. Moreover, imaging studies have revealed increased activation of brain regions associated with pain perception during painful stimuli in women with PVD [24], a pattern of results analogous to that observed in studies completed in other chronic pain populations such as fibromyalgia [25–27], irritable bowel syndrome [28–33], and idiopathic back pain [34].

Imaging studies have also shown structural changes in women suffering from PVD. Schweinhardt et al. [35] revealed higher gray matter densities in pain modulatory and stress-related areas in women with PVD, suggesting potential alterations in the supraspinal pain modulatory circuitry. These neuroanatomical changes in CNS regions related to endogenous pain modulation could explain the large-scale changes in pain sensitivity observed in women with PVD (e.g., lower pain thresholds in regions other than the vulvar vestibule). Another argument in favor of central pain mechanism alterations is the co-occurrence of other pain conditions such as orofacial pain, fibromyalgia, and irritable bowel syndrome [4, 36]. Arnold et al. [4] found that women with vulvar pain have a threefold to fourfold risk of having these concomitant pain conditions. Furthermore, similar medications (e.g., antidepressants) are commonly used to treat PVD, fibromyalgia, and irritable bowel syndrome, suggesting that these disorders may share similar pathophysiological mechanisms [37–39].

One proposed technique for modulating CNS activity in chronic pain states is noninvasive brain stimulation (NIBS). NIBS strategies aimed at modifying cortical excitability for different purposes have emerged in recent years [40]. Transcranial direct-current stimulation (tDCS) is a specific form of NIBS that has been shown effective for improving various chronic pain conditions relating to spinal cord injury [41], fibromyalgia [42], multiple sclerosis [43], painful diabetic polyneuropathy [44], pelvic pain [20, 45], and other various syndromes such as trigeminal neuralgia, post-stroke pain syndrome, and back pain [46]. tDCS is a painless technique that consists of applying a low direct current through electrodes placed on the scalp to target the cerebral cortex in order to modify cortical excitability and reduce pain [40]. tDCS is a safe and simple device and could be easily integrated into a rehabilitation program.

Even if a large literature concerning tDCS in pain relief now exists [40, 47, 48], to our knowledge, only one case looking into the effect of tDCS in women with vulvodynia [49] exists. In this case report, Cecilio et al. describe the case of a woman suffering from severe chronic vulvar pain refractory to many empirical treatments (tricyclic antidepressants, anticonvulsants, and opioid analgesics) who has experienced remarkable long-lasting pain relief with tDCS. On the basis of the pathophysiology of chronic pain related to PVD that is similar to other chronic pain syndromes, and considering that it is a neglected women’s health condition, we believe it is important to study the efficacy of this promising treatment in women with PVD. The main goal of this study is to evaluate the efficacy of active tDCS treatment in women with PVD compared to sham tDCS for pain during intercourse, as assessed with a numerical rating scale. We expect that active tDCS treatment will significantly reduce pain during intercourse (2-week post-treatment and 3-month follow-up compared to the pretreatment assessment). We expect that active tDCS treatment will be more effective for reducing pain than the sham tDCS treatment at 2-week post-treatment and 3-month follow-up.

Secondary objectives are to compare the efficacy of active tDCS vs. sham tDCS on sexual function, sexual satisfaction, vestibular pressure-pain threshold, psychological distress (catastrophizing, anxiety, depression, fear of pain, and vaginal penetration cognition), treatment satisfaction, and patient global impression of change (2-week post-treatment and 3-month follow-up compared to pretreatment assessment).

## Methods/design

### Design

**Fig. 1:**
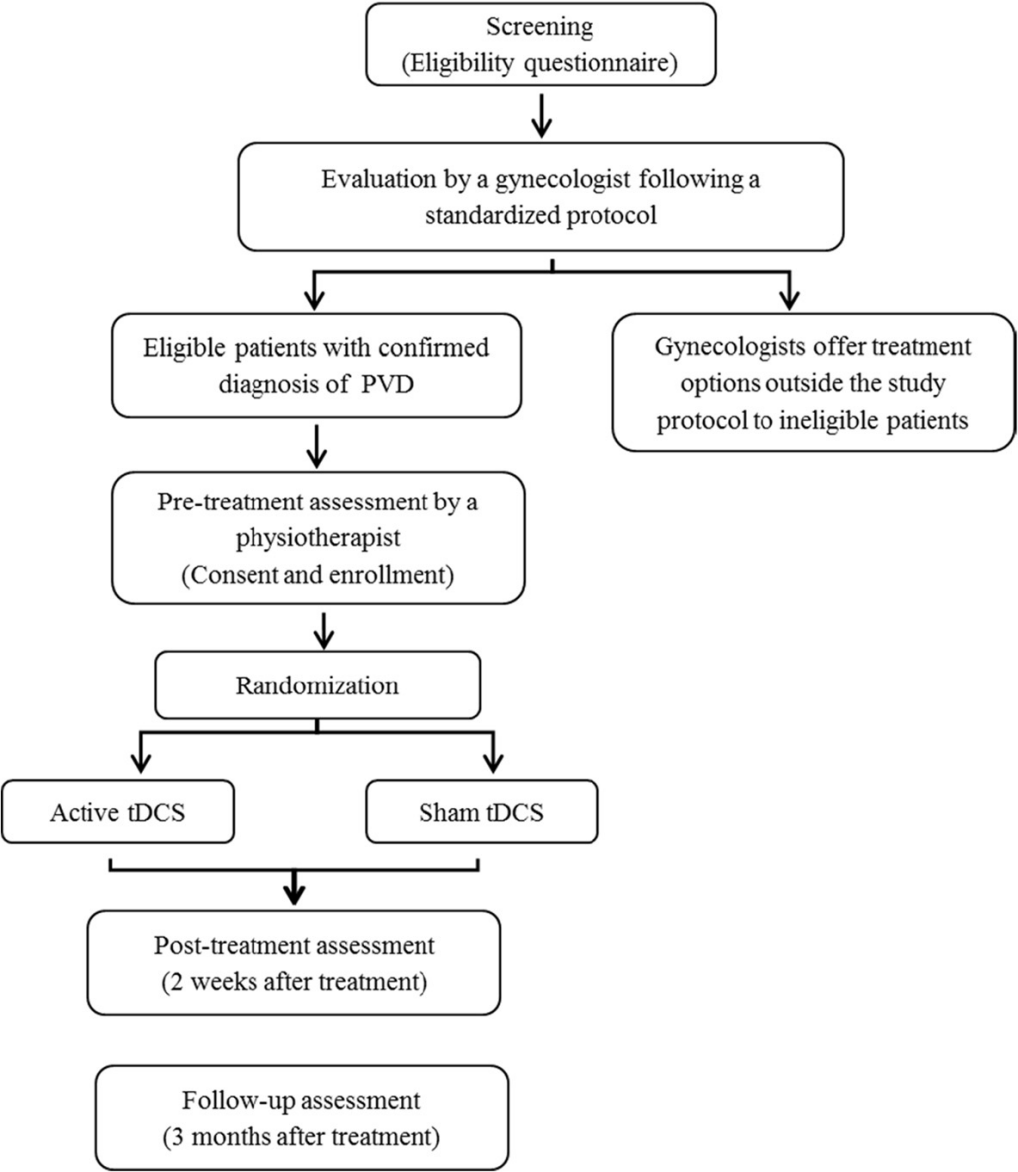
Study flow diagram

The proposed research design is a triple-blind (physiotherapist assessor, patient, and treatment provider), randomized, placebo-controlled, parallel-group trial. Participants are randomized to receive either active or sham tDCS for ten sessions of 20 minutes of stimulation over a 2-week period. As illustrated in the flow diagram (Fig. 1), the study includes three evaluation points (pretreatment assessment, post-treatment assessment performed at 2 weeks, and follow-up assessment at 3-months post-treatment). This study protocol was written in accordance with the SPIRIT statement (see Additional file 1 for the completed SPIRIT checklist).

### Participants

Premenopausal women, ages 18–45 years, suffering from pain during intercourse, are being recruited at the Research Center of the Centre Hospitalier Universitaire de Sherbrooke (CHUS). The eligibility criteria are detailed in Table 1. These criteria were selected to ensure recruitment of a homogenous sample of sexually active women with PVD and were based on previous successful studies [13, 50, 51]. In order to confirm the diagnosis of PVD, the eligibility screening comprises a gynecological assessment performed by a gynecologist from our team, following a standardized protocol. The latter consists of a brief anamnesis and a comprehensive gynecological examination of the vulvar region (clitoris, small lips, interlabial furrow, and vestibule), testing for vaginal infection and sexually transmitted disease, and palpation of the uterus and appendices. This evaluation follows the diagnostic criteria defined by Friedrich [2] and more recently modified by Bergeron et al. [52], namely, (1) pain in the vestibule following touch or an attempted vaginal penetration or (2) acute pain during palpation of the vestibule region with a cotton swab. Interevaluator fidelity of this diagnosis method has been demonstrated [52] and used in several RCTs [13, 53, 54]. Therefore, vulvar pain occurring in the absence of an underlying recognizable disease and provoked spontaneously as a result of physical contact can be classified as PVD [55].

**Table 1:** Eligibility criteria

*Inclusion criteria*	*Exclusion criteria*
- Experience moderate to severe pain (minimum of 5/10 on a numerical rating scale (NRS)) in at least 90 % of attempted sexual intercourse - Pain limited to the vestibule during vaginal intercourse and during activities exerting pressure on the vestibule (tampon insertion, tight jeans or pants, cycling, horseback riding) - Presence of provoked vestibulodynia (PVD) for at least 6 months and diagnosed according to the standardized gynecological examination protocol by one of our staff gynecologists - Have a stable sexual partner (sexual activity should include some attempted vaginal penetrations in order to evaluate pain intensity)	- Urogynecological conditions (e.g., active urinary tract or vaginal infection or in the last 3 months, etc.) and other pelvic pathology associated with pelvic pain (e.g., deep dyspareunia) - Have given birth in the last year and breast feeding - Anterior vulvar or vaginal surgery - Refusal to refrain from other treatments 1 month prior to first treatment study until the last 3-month follow-up assessment - Contraindications to transcranial direct-current stimulation (tDCS) (e.g., metallic implant in or near the skull, history of epilepsy, pacemaker) - Previously received tDCS treatment

### Treatments

Participants are randomized to receive ten sessions of either active or sham anodal tDCS over a period of 14 days. tDCS treatments are given once a day, during weekdays (Monday to Friday). Each session lasts 20 minutes [20, 41, 44, 56, 57] and is administered by a research professional experienced in tDCS. The treatment provider is not involved in the patient assessment and is blinded to the treatment allocation by selecting a preset program of the tDCS device (NeuroConn DC stimulator). Two electrodes are applied to the subject’s scalp; the anode is placed over the motor cortex (M1) [58], and the cathode, over the contralateral supraorbital area [20, 41, 44, 57, 58]. For treatment with active tDCS, the intensity of the stimulation is set at 2 mA for the entire duration of the treatment [20, 41, 42, 44, 49, 56]. The parameters used in our study have been tested with many subjects in several different laboratories without side effects [59], apart from a slight itching, tingling, or burning sensation under the electrode during the first seconds of stimulation; discomfort or erythema (skin reddening) under the active electrode; and possible headache in the hours following the treatment. If the stimulation is switched on or off abruptly, the sensation of a short ocular light flash could also be felt by the participant [60]. For subjects receiving sham tDCS (placebo group), the electrodes are positioned in the same areas as the group receiving active stimulation, and the intensity is set at 2 mA for the first 30 seconds of treatment [57], after which the stimulation stops automatically. Just as for active tDCS, participants are advised that a brief tingling sensation may be felt at the beginning of treatment. This method is effective for preserving subject and investigator blinding [61].

In order to report the participants’ adverse events during tDCS treatment, at each treatment session, the treatment provider notes the participants’ side effects, and the subjects are asked to complete a logbook in which they have to report whether they experience any adverse events or pain in the vulvar region, whether related to intercourse or not. Participants also have to record their experienced vulvar pain in the same logbook between the end-of-treatment period and the 2-week post-treatment assessment.

### Recruitment and procedures

Participants are recruited using different promotion strategies: (1) referrals from health professionals (gynecologist, general practitioner, sexologist, psychologist, or physiotherapist); (2) posters and leaflets distributed to women in clinics, universities, professional schools, restaurants, gyms, etc.; (3) Facebook advertisement; and 4) advertisements in local newspapers. Women interested in participating are invited to contact the research coordinator for a detailed explanation of the study and verification of their eligibility. Thereafter, participants undergo a gynecologist’s examination for confirmation of their diagnoses. Once a diagnosis of PVD has been confirmed, the research assistant contacts the participants to confirm their admissibility into the study. Upon doing so, she provides the participants with instructions to follow before the assessment and, then, fixes the appointments. To avoid effects on the vestibular pain sensitivity measurements prior to each assessment, participants will be asked to refrain from smoking and consuming caffeine (coffee, tea, and energy drinks) 4 hours prior to the evaluation [62], to refrain from taking painkillers (Advil®, Motrin®, Tylenol®, etc.) 24 hours before the evaluation [63], and to not have or attempt vaginal intercourse in the 24 hours before the evaluation [64]. After signing the informed consent, the eligible women are invited to undertake a 1-hour pretreatment assessment (baseline) with a physiotherapist specialized in pelvic-floor rehabilitation and pain assessment. During the pretreatment assessment, a structured interview is conducted to collect the patient’s baseline characteristics, and standardized questionnaires are completed. Approximately 30 minutes are required to complete all questionnaires. These questionnaires have been successfully used in our previous studies, without hindering data quality [65]. A vestibular pain sensitivity assessment is also undertaken using the algometer [66]. The participants are then randomized to receive either active or sham tDCS. The same procedures are repeated at the 2-week post-treatment and 3-month follow-up assessments.

### Participant retention and protocol adherence

As for other clinical trials, the greatest challenges pertain to participant retention. Strategies implemented to minimize attrition are as follows: (1) each participant is required to supply her contact information, such as phone number(s) and e-mail address; 2) a research assistant makes sure to remind participants of their appointments, as well as of the importance of their continuous participation; 3) all appointments are flexible to fit in with the participants’ schedule (i.e., early morning, late in the evening, etc.); and 4) participants receive a $20 compensation at three different stages of the study (the assessments) to cover travel expenses and their participation in the study. Adherence to the study protocol is also promoted throughout the trial. The physiotherapist-evaluator has received a standardized training to ensure homogeneity of procedures and reduce bias due to the evaluator. This physiotherapist-evaluator also has access to a detailed written assessment protocol Version 4 (Nov. 2, 2015). The research professional in charge of treatment is not involved in patient assessment and similarly has received proper training for the application of tDCS. The PI also carries out a weekly supervision of the physiotherapist’s and professional’s adherence to protocols. Moreover, the effectiveness of blinding for all physiotherapists and patients is evaluated with a questionnaire. Participants are also asked to abstain from the following PVD-related treatments (i.e., pelvic floor physical therapy, topical lidocaine application, vestibulectomy, and psychotherapeutic and systemic interventions) during their enrollment in the study, and this is monitored at the 2-week post-treatment and 3-month follow-up assessments. In order to avoid missing data, at the end of each assessment, the physiotherapist-evaluator revises all completed questionnaires before the participant leaves.

### Randomization and blinding

After the pretreatment assessment, the participants are randomized into either the active or sham tDCS treatment (ratio 1:1) using permuted blocks of two and four. The allocation is managed by an individual independent from our research team who follows a computer-generated randomization list drawn up by an independent statistician. Participants, investigators, physiotherapist assessors, and the treatment provider remain blinded to group allocation and, therefore, cannot influence the process in any way.

### Outcomes

The Initiative on Methods, Measurement, and Pain Assessment in Clinical Trials (IMMPACT) has recently recommended several outcome domains for use in evaluating the effectiveness of treatment in chronic pain conditions [67]. The current protocol adheres to these recommendations, with outcomes focusing on the multiple dimensions of PVD, such as pain, sexual function, psychological distress, participant ratings of improvement and satisfaction with treatment, and the need to assess the potential impact of treatment on these different dimensions.

#### Primary outcome measure

As recommended by the IMMPACT [67, 68], participants are asked to evaluate their pain during intercourse with an NRS, 0 indicating no pain, and 10, the worst pain ever experienced. This scale has been frequently used in women with PVD [13, 69–71] and shows good reliability, validity, and responsiveness to change [13, 52, 69–71].

#### Secondary outcome measure

##### Pain measurements

Different measurements of pain are recommended by the IMMPACT consensus group in order to evaluate different domains of pain [67, 72]. Ranging from 0 to 74, the McGill-Melzack questionnaire is a 20-item scale allowing the assessment of pain quality (i.e., sensory, affective, and evaluative components). A higher total score is related to more severe pain. This world-renowned questionnaire, studied for its validity, reliability, and responsiveness to change, is commonly used in RCTs [13, 52, 73–78].

Furthermore, the third International Consultation on Sexual Medicine has emphasized the importance of assessing vestibular sensitivity [79]. Our laboratory recently developed an algometer to measure the vulvar pain threshold and tolerance in women with PVD. A gradual pressure (1 to 1000 grams) is applied to three distinct points of the vestibule at the 3, 6, and 9 o’clock positions [80]. Each of these pressure points is applied randomly (e.g., 3,6,9 or 3,9,6 or 6,9,3). During this procedure, each participant is asked to indicate when they start to feel pain (pain threshold) and subsequently the maximal pressure they can tolerate (pain tolerance) (see [66] for more details). Pain intensity is assessed throughout the test using a computerized visual analog scale (CoVas). This assessment has shown good reliability and validity [80].

##### Sexual function measurements

The Female Sexual Function Index (FSFI) is a 19-item multidimensional measure of sexual function evaluating desire, arousal, lubrication, orgasm, satisfaction, and pain. This questionnaire is frequently used in trials involving women with PVD [13, 78]. In addition to good psychometric properties (reliability, internal consistency, and responsiveness to change) [8, 81], normative data are available for this questionnaire, suggesting clinical levels of dysfunctions [82]. Women considered at risk for sexual dysfunction record a FSFI total score of 26 or less [82].

In order to measure the participant’s sexual satisfaction, the Global Measure of Sexual Satisfaction (GMSS) questionnaire is used. This five-item questionnaire has a total score ranging from 5 to 35 and presents good psychometric properties (a high level of internal consistency and test-retest reliability) [83]. Better sexual satisfaction will result in a higher total score. Inclusion of these questionnaires is in line with the recommendations of the IMMPACT consensus group [67] recommending evaluation of the impact of pain on functions.

##### Psychological distress

Many studies have also determined that vulvodynia may even be a source of psychological distress [5, 84]. For these important reasons, different psychological variables are evaluated.

The Pain Catastrophizing Scale (PCS) is a 13-item questionnaire with good reliability, consistency, and responsiveness [8, 85] for evaluating the level of pain catastrophization, a robust predictor of pain and incapacity. The PCS yields a total score (0–52) and three subscale scores assessing rumination (0–16), magnification (0–12), and helplessness (0–24). Research at the University Centre for Research on Pain and Disability indicates that a total PCS score of 30 represents a clinically relevant level of catastrophizing [86].

The State-Trait Anxiety Inventory of Spielberger (STAI-Y) allows discrimination between anxiety as a trait of personality (T-Anxiety) or as an emotional response to a situation (S-Anxiety) [87]. This 40-item questionnaire, in which 20 items are allocated to each of the S-Anxiety and T-Anxiety subscales, has previously been used in women with PVD [78] and has shown a good reliability, consistency, and responsiveness [8, 87]. Responses for the S-anxiety scale assess intensity of current feelings “at this moment” according to the following: (1) not at all, (2) somewhat, (3) moderately so, and (4) very much so. Responses for the T-anxiety scale assess frequency of feelings “in general” according to the following: (1) almost never, (2) sometimes, (3) often, and (4) almost always [88]. Although this questionnaire was not designed or validated to be used with a cut-off score, an earlier Hungarian research used T-anxiety cut-off values as follows: less than 48 indicating no anxiety, 48 to 52 indicating mild or sub-clinical disorder, and higher than 52 indicating significant anxiety [89].

The Beck Depression Inventory questionnaire (BDI) [90] was recommended by Dworkin et al. [67] for assessing depression [91]. This scale has been studied for its reliability and consistency [91] and has also been used in women with PVD [78]. This 21-item questionnaire has a total score ranging from 0 to 63. Higher total scores indicate more severe depressive symptoms. Total score cut-offs for this questionnaire are as follows: 0–9 indicating that a person is not depressed, 10–18 indicating mild-moderate depression, 19–29 indicating moderate-severe depression, and 30–63 signifying severe depression [90].

The Pain Anxiety Symptoms Scale (PASS-20), which evaluates four distinct components of pain-related anxiety (i.e., cognitive, fear, escape/avoidance, and physiological), also shows good psychometric properties [92, 93]. The total score of this 20-item questionnaire ranges from 0 to 100. A higher total score is related to higher levels of pain-related anxiety. Individuals with acute pain presenting a total score exceeding 30 may be at an elevated risk for maladaptive pain cognitions and behaviors promoting chronic pain and disability [94].

##### Satisfaction with treatment and the Patient’s Global Impression of Change

The dimension of patient’s global impression of change (PGIC) is also evaluated [95, 96]. PGIC is a validated questionnaire [97] through which patients self-report selected measures to evaluate perceived reduction in pain using a seven-point scale ranging from “very much improved” to “very much worse” and rate their treatment satisfaction over an 11-point scale that ranges from “completely satisfied” to “completely dissatisfied” in order to assess the clinical significance of the results. Subjective evaluations of pain improvement and treatment satisfaction have previously been evaluated in a randomized trial involving women with PVD [13].

### Ethical aspects

The study has received ethical approval from the comité d’éthique de la recherche en santé chez l’humain du CHUS (14-169). Every potential participant is made aware that there will be no impact on her medical care if she decides not to participate in the study. Women who refuse to participate in the study or who do not fulfill the eligibility criteria are followed by the clinical staff using the most recently proposed vulvodynia guidelines. In order to minimize privacy risks, all participants are identified by an alpha-numeric code. Informed consent is obtained from all participants prior to trial inclusion. This trial is registered at clinicaltrials.gov NCT02543593.

### Statistical considerations

#### Sample size, power, and statistical analysis methods

A total sample size of 34 participants is sufficient to detect a clinical minimal significant difference of 2 [67, 98] on an NRS (unpaired *t* test; α = 0.05; β = 0.80, standard deviation of 2.0) based on the efficacy of tDCS in other painful conditions [57, 99]. This estimation is conservative considering that tDCS in chronic pain demonstrated a mean pain reduction of 4.34 points [40] and that the available case study in a woman with vulvodynia showed a reduction of ten points [49]. To account for potential dropouts, a total of 40 subjects are being recruited. This estimated dropout rate (< 15 %) is based on available studies and our own RCT experience in women with PVD [13, 78, 100].

#### Statistical analyses

Baseline characteristics of the sample will be presented using descriptive statistics. Analyses will be done on the intention-to-treat basis. Parametric test assumptions will be examined and reported. If the data is not normally distributed, nonparametric data will be transformed. The effects of treatment on pain, sexual function and psychological distress will be examined using a mixed linear model for repeated measures. One of the factors will be the GROUP, at two levels (the treatment group receives active tDCS, and the control group, sham tDCS), while the repeated factor will be TIME (baseline, 2-week post-treatment, and 3-month follow-up assessments). Treatment efficacy will be judged on the basis of a significant GROUP*TIME interaction and contrast analysis (i.e., Tukey-Kramer contrasts when using a mixed-model approach) [101] to detect any differences. The difference between the two groups regarding satisfaction and PGIC will also be assessed using a mixed linear model for repeated measures. However, the baseline assessment will not be a repeated time-factor for these analyses. All statistical analyses will be conducted at a level of significance of 0.05. Sensitivity analyses will be conducted to explore the effect of multiple imputation methods to replace missing data. The non-robustness of our results due to missing value will be noted and discussed.

## Discussion

This is the first study to evaluate the efficacy of tDCS for reducing pain during intercourse in women with PVD. Considering that the current literature on PVD pathophysiology has converged toward the cause being central pain sensitization, tDCS is a promising treatment targeting central mechanisms rather than focusing strictly on the vestibule.

The plausibility of treatment effects relies on a large body of evidence supporting treatment efficacy in various chronic pain conditions [40], including a case study showing complete pain resolution in a woman with vulvodynia [49]. The underlying mechanisms of action also support the potential short and long-lasting effects in women with PVD. Immediate effects of tDCS are explained by polarity-dependent shifts of the resting membrane potential and consequent alteration of corticospinal excitability at the stimulation site. Alteration leads to facilitation or inhibition of the superficial structures and of deeper and more remote brain areas related to pain modulation, such as the periaqueducal gray, insula, and thalamus [40]. Thus, the immediate effects of tDCS are a consequence of neuronal hyperexcitability caused by the anode or hypoexcitability induced by the cathode [47], whereas the long-lasting effects of tDCS seem to depend on N-methyl-D-aspartate (NMDA) receptor-efficacy changes [102]. In patients with chronic pain, the anode is commonly placed over the motor cortex (M1) [103]. According to functional imaging studies, stimulation of the motor cortex modulates activity in the limbic system, brainstem and spinal cord, which are all involved in the emotional component of pain [104–106]. Therefore, even if the genital zone is located deeper in the central sulcus of the somato-sensorial cortex, available evidence suggests that the mechanism of action of tDCS for reducing pain involves the emotional component of pain.

This randomized placebo-controlled trial addresses the urgent need to provide evidence-based treatments for women with PVD. In addition, blinding of participants, evaluators, and investigators, as well as the use of recommended validated tools, strengthened our study design. Given the efficacy found in various chronic pain conditions and the promising results obtained in a case report study in a woman with vulvodynia, this new treatment avenue may give hope to women who have experienced failure with the available localized treatments.

### Trial status

The recruitment of participants is ongoing at the Research Center of the CHUS. Forty women with PVD are planned to be recruited and randomized from December 2014 to May 2016.

## Additional file

Additional file 1: SPIRIT checklist. Completed SPIRIT checklist. (PDF 51 kb)

## Abbreviations

ANOVA: analysis of variance
BDI: Beck depression inventory questionnaire
CBT: cognitive behavioral therapy
CHUS: Centre Hospitalier Universitaire de Sherbrooke
CNS: central nervous system
CoVas: computerized visual analog scale
FSFI: female sexual function index
GMSS: global measure of sexual satisfaction
IASTA: state-trait anxiety inventory of Spielberger
IMMPACT: Initiative on Methods, Measurement, and Pain Assessment in Clinical Trials
M1: motor cortex
mA: milliamperes
MPQ: McGill pain questionnaire
NIBS: non-invasive brain stimulation
NMDA: N-methyl-D-aspartate
NRS: numeric rating scale
PASS-20: pain anxiety symptoms scale
PCS: pain catastrophizing scale
PGIC: patient’s global impression of change
PVD: provoked vestibulodynia
RCT: randomized controlled trial
SSRIs: selective serotonin reuptake inhibitors
tDCS: transcranial direct-current stimulation
